# Identification and Characterization of the Grape WRKY Family

**DOI:** 10.1155/2014/787680

**Published:** 2014-04-27

**Authors:** Ying Zhang, Jian can Feng

**Affiliations:** Forestry Department, Agricultural University of Henan Province, Zhengzhou, Henan 450002, China

## Abstract

WRKY transcription factors have functions in plant growth and development and in response to biotic and abiotic stresses. Many studies have focused on functional identification of WRKY transcription factors, but little is known about the molecular phylogeny or global expression patterns of the complete WRKY family. In this study, we identified 80 WRKY proteins encoded in the grape genome. Based on the structural features of these proteins, the grape *WRKY* genes were classified into three groups (groups 1–3). Analysis of *WRKY* genes expression profiles indicated that 28 *WRKY* genes were differentially expressed in response to biotic stress caused by grape whiterot and/or salicylic acid (SA). In that 16 *WRKY* genes upregulated both by whiterot pathogenic bacteria and SA. The results indicated that 16 WRKY proteins participated in SA-dependent defense signal pathway. This study provides a basis for cloning genes with specific functions from grape.

## 1. Introduction

Various defense mechanisms have evolved in plants to combat microbial infection. Transcription factors are central to this process, and transcription factor families have expanded and evolved in plants to coordinate gene expression. Expression of a large number of defense-related plant genes is regulated at the transcriptional level in response to pathogen infection [1]. Timely transcriptional regulation of defense-related genes is crucial for effective responses to pathogens [2]. Proteins of the WRKY family are the most important transcription factors for the regulation of plant defense response pathways [3–5]. The WRKY name is derived from the conserved DNA binding domain sequence WRKYGQK; the conservative domain is approximately 60 residues, followed by a Cys2His2 or Cys2HisCys zinc-binding motif [6, 7], and WRKY proteins were divided into Group I-III based on the number of WRKY domains and the structure of zinc fingers [8, 9]. The Group II WRKY proteins are classified into a, b, c, d, and e subgroups based on their zinc finger motifs [5].

In higher plants, the* WRKY* gene family members play a variety of roles. Accumulating evidence indicates that WRKY transcription factors are involved in responses to biotic stresses as well as in plant development [7, 10, 11]. Salicylic acid (SA) is an response endogenous phytohormone and an important signal substances in the deployment of systemic acquired resistance (SAR) [12]. SAR is characterized by an increase in endogenous salicylic acid (SA) and enhanced resistance to a broad spectrum of virulent pathogens. SA is necessary for SAR, and a series of studies demonstrated that SA triggers host defence mechanisms against pathogen infections [13, 14]. Many WRKY genes are key factors controlling plant response to disease resistance especially pathogen infections that can trigger SA-dependent defense signaling. In* Arabidopsis,* WRKY70 was identified as an important node of SA signaling during plant defense responses [15]. In addition, treatment of* Arabidopsis* with a bacterial pathogen or salicylic acid (SA) resulted in differential expression of WRKY genes [15, 16]. This pattern of expression was also observed in other plant species [10, 17–19].

Cultivated grapevines are susceptible to many pathogens including phytoplasmas, viruses, bacteria, and fungi [22]. Among these, grape white rot (*Coniothyrium diplodiella*) is the most important agriculturally because it causes extensive losses in quantity and quality of harvested berries. As a consequence, table and wine grape cultivation requires extensive use of phytochemicals. In China, grape white rot is the main fungal disease of grapes causing heavy losses in grape production [23].* Vitis vinifera *L. (“European” grape) is the most economically important and widespread species of* Vitis* spp. producing more than 90% of world's production of table, wine, and raisin grapes. More than 80% of cultivated grape varieties are derived from this species. All* V. vinifera *L. varieties are susceptible to grape white rot [24]. The disease is found in most grape-growing regions of the world [25] and results in poor berry quality and weakened vines in warm and humid climates. Multiple research strategies are currently being pursued so that wine growers may produce healthy fruits right up to maturity with minimum use of chemical treatments.

The complete* V. vinifera* PN40024 genomic sequence [26, 27] has been determined. The* V. vinifera* genome was the first fruit tree species genome to be sequenced, making* V. vinifera* an ideal model system for fruit trees [26]. The release of the latest* V. vinifera *genome sequence, 12X (http://www.genoscope.cns.fr/externe/GenomeBrowser/Vitis/), and the many WRKY gene and protein sequences from other* Vitis *spp. present in the NCBI (http://www.ncbi.nlm.nih.gov/) and PlantTFDB (http://planttfdb.cbi.pku.edu.cn/index.php?sp=Vvi) databases provided an opportunity to analyze and further understand the grape* WRKY* gene family. In this study, we identified a comprehensive and nonredundant set of eighty* WRKY* genes in the grape genome. Phylogenetic and motif analysis and characterization of WRKY expression induced by pathogens and salicylic acid (SA) were also performed to lay a solid foundation for further comparative genomics studies.

## 2. Materials and Methods

### 2.1. Characterization of Putative WRKY Proteins in Grape

The latest 12X* V. vinifera* genomic and protein sequences were downloaded from the NCBI database. The procedure used to survey grape WRKY proteins was similar to identification methods described for other species. The hidden Markov model (HMM) profile for the WRKY domain from the Pfam database was used as a query to survey all potential proteins. The Pfam database was then used to decide if the candidate proteins contained features typical of WRKY proteins. Identical and defective sequences were eliminated using manual inspection in the MEGA ver4.0 software. Nonoverlapping WRKY protein sequences were used for further analysis.

### 2.2. Phylogenetic Analysis Based on Conserved WRKY Domains

WRKY genes of was retrieved by TBlastN software from the publicly available information in the database (http://www.ncbi.nlm.nih.gov, http://www.genoscope.cns.fr/externe/GenomeBrowser/Vitis/). Conserved WRKY domains of the VvWRKY proteins were identified by manual inspection using the Pfam software and used to generate a multiple sequence alignment of the WRKY domains. Phylogenetic trees based on 58 representative domains from poplar, Arabidopsis, rice, and grape were constructed using Clustal X ver1.83 and Mega ver4.0 [28] by the neighbor-joining (NJ) method to produce improved classifications of the different clades. Bootstrap values were calculated from 1,000 iterations.

### 2.3. Analysis of Conserved Motif Distribution and Structure and Gene Duplication in Grape

To assess the structural divergence of* VvWRKY* genes, conserved motifs in the encoded proteins were identified using the multiple expectation maximization for motif elicitation (MEME) online software (http://meme.sdsc.edu/meme/ intro.html). Parameters employed in the analysis were as follows: maximum number of motifs, 20; minimum motif width, 6; and maximum motif width, 50. The online software 2ZIP (http://2zip.molgen.mpg.de/index.html) was used to predict the conserved Leu zipper motif. HARF, LXXLL, and LXLXLX motifs were identified manually.

### 2.4. Expression Analysis

Chinese wild grape* V. vinifera *“Pinor” leaves (numbered by the National Repository for Grapevine (Zhengzhou grape germplasm repository)) were used for all experiments. When shoots of vines were 25–35 cm in length, the third through fifth fully expanded young leaves beneath the apex were selected for biotic and abiotic treatments. The plants were subjected to abiotic stress and SA treatments according to Ramamoorthy et al. [29]. For SA treatment, plants were sprayed with a 0.1 mM SA solution. Treatment with deionized water served as a control and was repeated three times on three independent plants. Leaves were collected 0, 9, 12, 24, and 48 h after treatment and immediately frozen in liquid nitrogen for further study.

For pathogen treatment, leaves were inoculated with* Coniothyrium diplodiella* mycelium gelose discs from a 3-day-old culture at six sites and placed on PDA medium at 28°C. Leaves sprayed with PDA medium were used as negative control. Leaves were collected 0, 9, 12, 24, and 48 h after inoculation (hpi). Treatment with deionized water was performed as a control. After harvest, the materials were immediately frozen in liquid nitrogen and stored at −80°C for further analysis.

Grape total RNA was extracted as previously described [30]. Grapevine total RNA was extracted from* V. vinifera *“Pinor” leaves using an improved SDS/phenol method [31] at 0, 9, 12, 24, and 48 h after infection with* Coniothyrium diplodiella*. The following primers were used for RT-PCR amplification: 5′-GCGGGCAAGAGATACCTCAA-3′ and 5′-TCAATCTGTCTAGGAAAGGAAG-3′ for* EF1*γ** (AF176496). Three independent PCR reactions were carried out for each gene and similar results were obtained. Amplification products were quantified using a Roche 480 II real-time PCR instrument.

## 3. Results

### 3.1. Identification of WRKY Proteins from Grape

To identify WRKY proteins encoded in the grape genome, publicly available genome sequences were searched using the BlastP software based on an HMM (PF03106). A total of 80 putative grape WRKY protein sequences were initially identified. With the exception of VvWRKY1, VvWRKY2, VpWRKY1, and VpWRKY2, none of the proteins were described previously. Based on manual inspection using the MEGA ver4.0 software, seven sequences were discarded due to redundancy or insufficient conservation of WRKY sequences. The remaining WRKY proteins were reviewed using the Pfam program to confirm that all candidates contained the conserved WRKY motif. Properties of the proteins including numbers of amino acids, molecular weights, and isoelectric points (PI) are listed in Table 1. The average VvWRKY sequence length was 382 amino acids and lengths ranged from 151 (VvWRKY1-1) to 798 (VvWRKY9) residues, while the isoelectric points (PI) ranged from 4.7 (VvWRKY22-4) to 9.84 (VvWRKY21).

**Figure 1:**
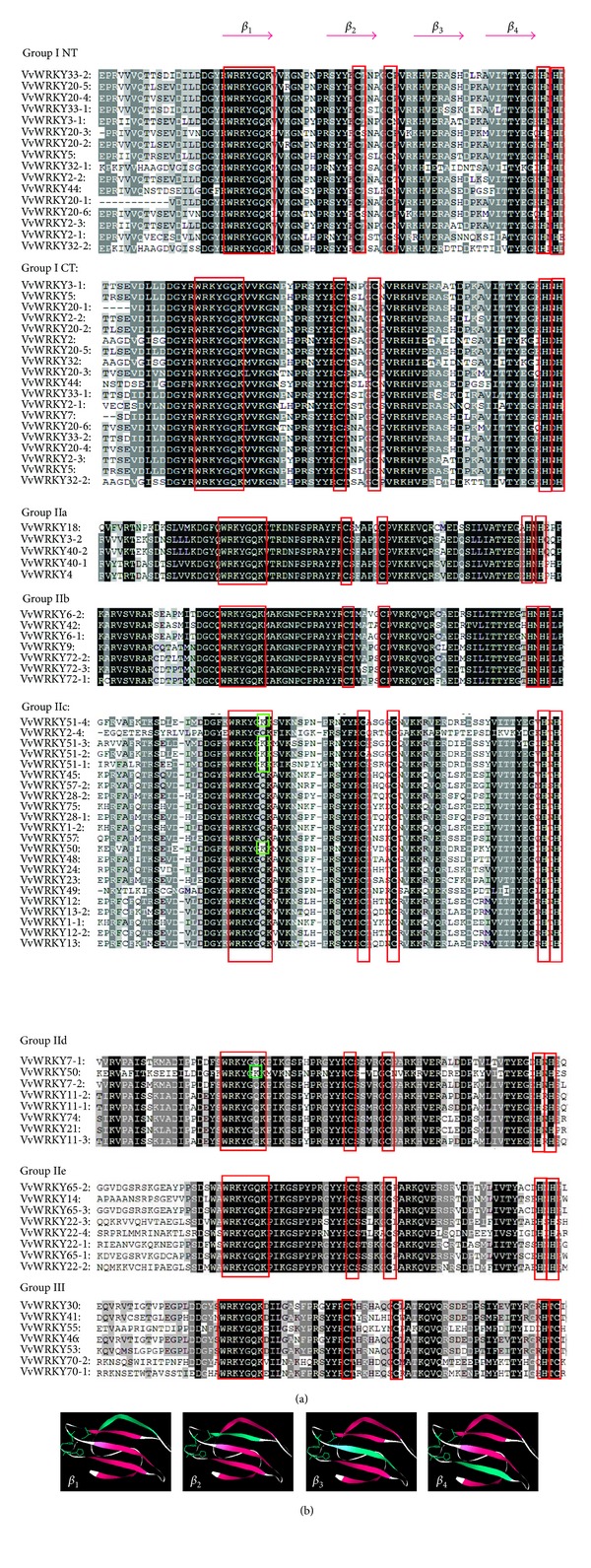
The WRKY conservative domain. (a) Comparison of WRKY domain sequences from VvWRKY proteins. Sequences encoding the peptide stretch WRKYGQK were found by the BLAST programs tblastn and blastp programs in genomic and EST databases. Gaps (dots) have been inserted for optimal alignment. Residues that are highly conserved within each of the major groups are in black and potential zinc ligands are highlighted in red boxes, and the different amino acid residues are highlighted in green boxes. (b) The four *β*-strands are shown in red. I CT and I NT denote the N- and C-terminal WRKY domains from Group I WRKY proteins. As in (b), the *β*_1_, *β*_2_, *β*_3_, and *β*_4_ motifs are shown in green and the Zn finger is indicated by a green line.

The WRKY domain is approximately 60 amino acid residues in length and is considered to be a crucial element for interaction with the W-box (C/T)TGAC(T/C) to activate many defense-related genes. In our study, 80 WRKY domains contained highly conserved WRKYGQK sequences, while the other WRKY domains had one amino acid mismatch in the conserved WRKY sequence (Figure 1). In VvWRKY51-2, 51-3, and 51-3, the WRKY domain sequence was WRKYGKK. As described by Eulgem et al. [7], the metal-chelating zinc finger motif (C-X_4-5_-X_22-23_-H-X-H or C-X_5–8_-C-X_25–28_-H-X_1-2_-C) is another important characteristic of WRKY proteins. Zinc-finger-like motifs were identified in all of the grape WRKY proteins.

### 3.2. Phylogenetic Analysis and Classification of WRKY Groups in Grape

A phylogenetic tree was constructed based on the conserved WRKY domains to examine the phylogenetic relationships among all 80 members. VvWRKY domains of the Group I include two domains (the C-terminal and N-terminal domain). A multiple sequence alignment of the 80 WRKY domains was performed (Figure 1). Three major groups were identified as described by Wu et al. [20]. In addition, several subgroups were revealed by the phylogenetic analysis.

**Figure 2:**
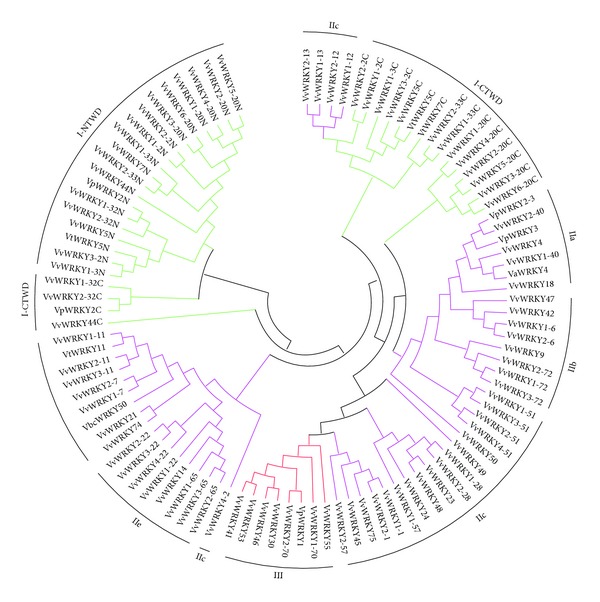
Phylogenetic tree based on amino acid sequences as determined by the MEGA ver4.0 software using the neighbor-joining method. Bootstrap values (≥500) based on 1,000 replications are exhibited beside the nodes.

**Figure 3:**
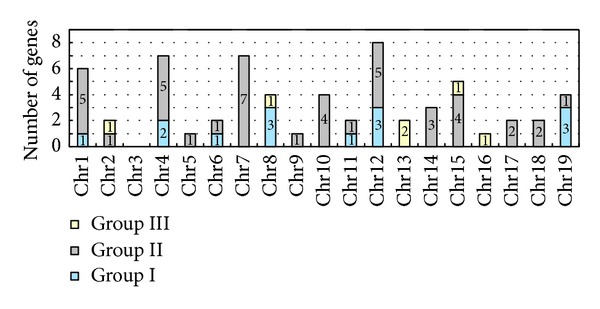
Histogram of the number and distribution of three groups of WRKY genes on 19 chromosomes.

The positions of the C- and N-terminal WRKY domains in the WRKY proteins were relatively consistent. Group I contained 19 WRKY proteins all of which contained two WRKY domains. Three Group I-CTWD (C-terminal domain) members, VvWRKY32-1C, VvWRKY32-2C, and VpWRKY2C, were more closely related to the N-terminal members of Group I than to the other C-terminal members (Figures 2 and 3). While Group I VvWRKY44C was far away from both I-CTWD and N-terminal, and unique, which suggesting a special origin of the domain. The largest number of WRKY proteins of Group II was divided to five among 23 major subgroups: IIa, IIb, IIc, IId, and IIe. Group IIa (seven members) and Group IIb (eight members) were two subgroups from the same branch, while Group IId (nine members) and Group IIe (eight members) were close in genetic tree. Five members of Group IIc were more similar to Groups IIa and IIb from the same branch based on the phylogenetic analysis. Four members of Group IIc, VvWRKY13-1, 13-2, 12-1, and 12-2, were more closely related to the C-terminal WRKY domains of Group I than those of other groups. This is consistent with a recent analysis supporting the hypothesis that loss of the Group I N-terminal WRKY domain led to the origin of the Group II WRKY proteins [5].

**Figure 4:**
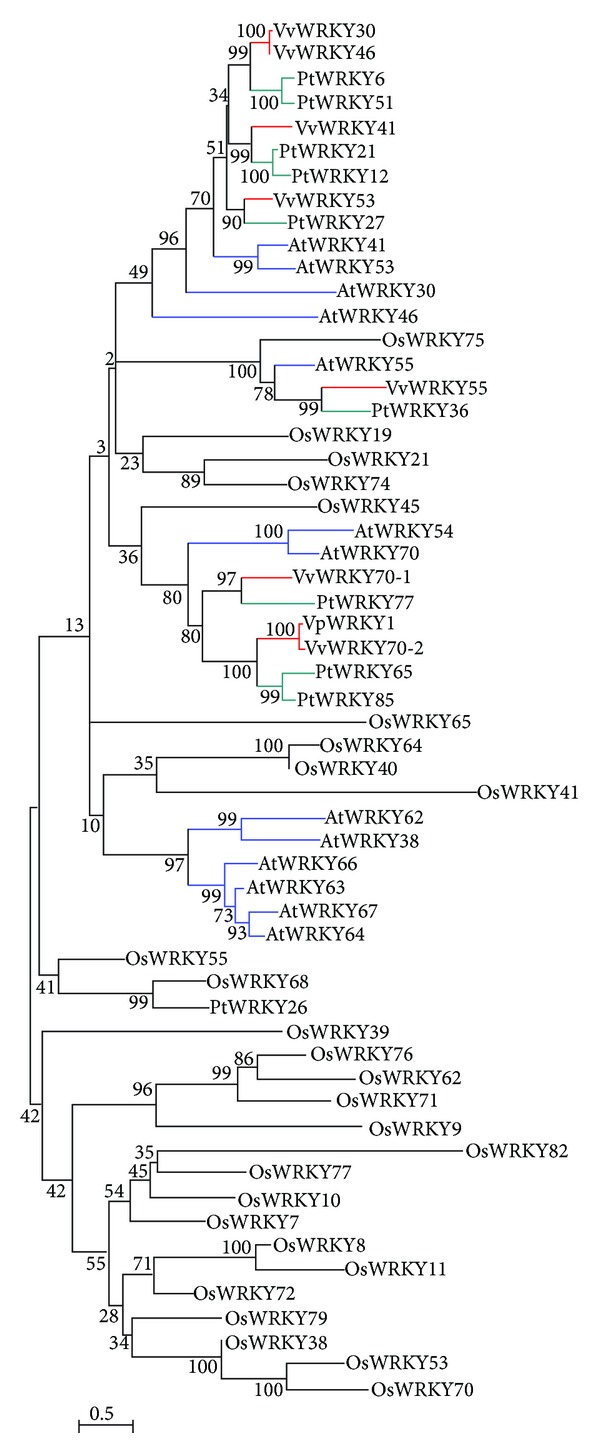
Phylogram of Group III WRKY domains from* Arabidopsis* (AtWRKY), rice (OsWRKY), poplar (PtWRKY), and grape (VvWRKY and VpWRKY). The alignment of amino acid sequences was produced using the MEGA ver4.0 program with the neighbor-joining method.

Seven WRKY domains belonged to Group III, which is generally considered to be the most evolutionarily advanced group and the most adaptable [5]. Phylogenetic relationships between the Group III WRKY domains of 15 AtWRKY, 28 OsWRKY, 10 PtWRKY, and 7 VvWRKY proteins were examined (Figure 4). The poplar WRKY domains shared higher sequence homology with the other dicot plants (*Arabidopsis* and grape) than with monocot rice. Similarly, the six AtWRKY domains clustered in two groups (Figure 4). However, this diversity was not found in* Arabidopsis*, poplar, or grape, suggesting that VvWRKY genes respond to different environmental signals.

### 3.3. Conserved Motifs and Domains in Grape WRKY Proteins

**Figure 5:**
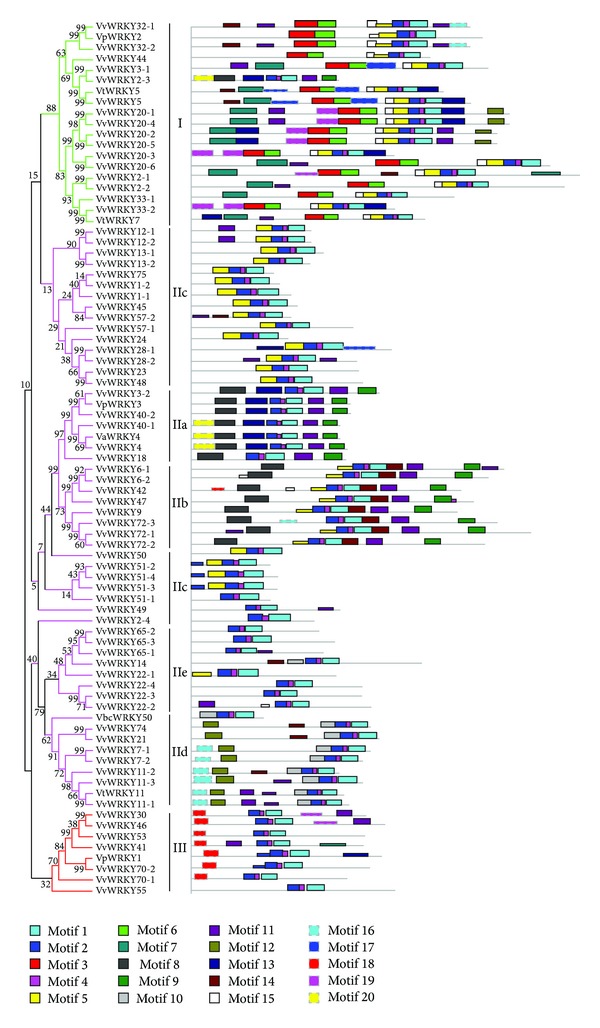
Phylogenetic analysis of 80 members of the* Vv*WRKY family. Amino acid sequences from the single WRKY domain of Groups II and III proteins or the C-terminal WRKY domain of Group I proteins were aligned. Conserved primary structure features of the* Vv*WRKY family located outside the WRKY domains were identified using MEME software (http://www.sdsc.edu/MEME) and are shown below the tree. Schematic representations of typical proteins of each (sub) group are shown on the bottom; motifs of amino acid sequences were shown in Table 2; LZ indicates potential leucine zipper structures that were also predicted by the COILSCAN and COIL (Wisconsin Package Version 10.0) programs.

With the exception of the conserved 60 amino acid residues, no functional or structural homologies were present in the WRKY protein sequences [7]. Few WRKY proteins contain a conserved leucine zipper motif, a hypothetical structure common to a class of DNA-binding proteins [7, 32]. Using the online 2ZIP software, we found that none of the grape WRKY proteins contained this structure with the exception of VvWRKY18. The conserved HARF sequence motif (RTGHARFRR(A/G)P) was found in six members (VvWRKY7-1, -7-2, -11-1, -11-2, -11-3, and VtWRKY11) of the Group IId WRKY proteins in* Arabidopsis*, although no putative function has been clearly identified for this motif [7, 33]. WRKY proteins are transcription factors associated with activation and repression of plant immune responses [7, 10, 34]. The coactivator motif, LXXLL (L, leucine; X, any amino acid), and the active repressor motif, LXLXLX [35, 36], were searched for in the VvWRKY protein sequences by manual inspection. Seven VvWRKY proteins, VpWRKY3 and VvWRKY20-3, -42, -9, -18, -3-2, and -40-2, contained the LXLXLX motif and three VvWRKY proteins (VvWRKY7-2, -11-2, and 11-3) contained the LXXLL motif. The Multiple expectation maximization for motif elicitation online software was used as a secondary method to analyze motif distribution and confirm the results of domain prediction (Figure 5; Table 2). LZ indicates potential leucine zipper structures that were also predicted by the COILSCAN and COIL programs but none was found. The conserved motifs 1, 2, 3, 4, 5, and 6 in Figure 5 were characterized as WRKY domains and were broadly distributed among the VxWRKY protein sequences. Motif 10 and motif 13, a conserved NLS motif, were found mainly among the Groups I and IId proteins, while motif 12 was found only among Groups I and IId proteins, although its function is unknown.

### 3.4. Quantitative RT-PCR Analysis of Expression of 28 WRKY Genes

**Figure 6:**
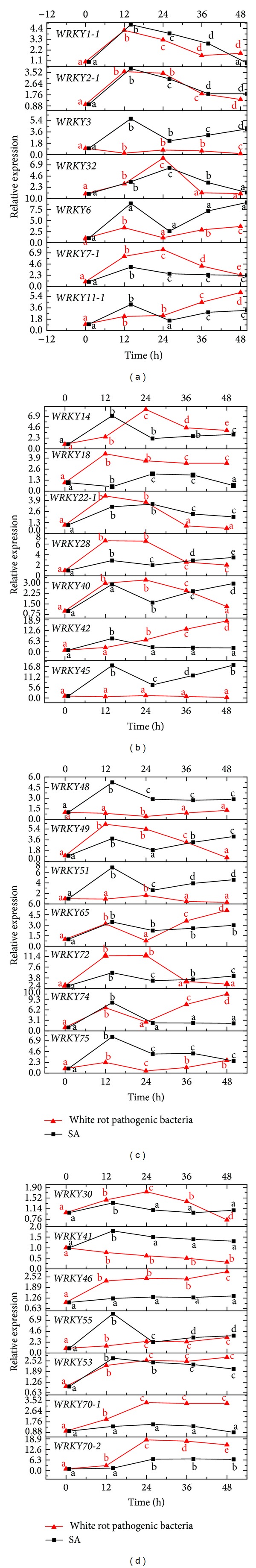
Quantitative RT-PCR analysis of VdWRKY subgroup gene expression in response to pathogenic fungal infection and SA treatment. 0 h (control) indicates treatment with deionized water. Grape EF**γ** gene (accession number AF176496) expression served as an internal control.

To analyze expression patterns of* WRKY* genes during plant defense responses, we analyzed the expression profiles of 28* WRKY* genes under disease conditions and in response to SA treatment using quantitative RT-PCR (Figure 6). Many of the* WRKY* genes exhibited significant changes in their expression levels in response to disease or SA. We isolated total RNA from leaves at various time points after infection with the fungal pathogen* Coniothyrium diplodiella* or after treatment with SA. The expression patterns of the 28* WRKY* genes (*VvWRKY1-1*,* VvWRKY2-1*,* VvWRKY3*,* VvWRKY6-1*,* VvWRKY7-1*,* VvWRKY11-4*,* VvWRKY14*,* VvWRKY18*,* VvWRKY22-1*,* VvWRKY28*,* VvWRKY30*,* VvWRKY32*,* VvWRKY40*,* VvWRKY41*,* VvWRKY42*,* VvWRKY45*,* VvWRVvKY46*,* VvWRKY48*,* VvWRKY51*,* VvWRKY53*,* VvWRKY55*,* VvWRKY65*,* VvWRKY70-1*,* VvWRKY70-2*,* WRKY72, *and* VvWRKY74*) were determined. After pathogen infection, three* WRKY* genes (*VvWRKY48*,* VvWRKY51*, and* VvWRKY45*) showed little or no change in transcript levels, but the other 25 genes showed altered expression patterns. Among the 25 differentially regulated genes, the expression of two (*VvWRKY3* and* VvWRKY41*) was repressed and that of 23 was induced. After SA treatment, 5 genes (*VvWRKY41, VvWRKY30, VvWRKY42, VvWRKY46, *and* VvWRKY70-1*) showed little or no change in transcript levels, but expression of the other 23 genes was induced and upregulated. 16 of 23 (*VvWRKY1-1, VvWRKY2-1, VvWRKY3, VvWRKY6-1, VvWRKY7-1, VvWRKY11-4, VvWRKY14, VvWRKY22-1, VvWRKY28, VvWRKY32, VvWRKY40, VvWRKY53, VvWRKY55, VvWRKY65, VvWRKY70-1,* Vv*WRKY70-2, WRKY72, *and* VvWRKY74*) upregulated* VvWRKY* genes were induced and upregulated by both pathogen infection and SA treatment (Figure 6).

## 4. Discussion

The WRKY transcription factor gene family appears to be involved in the regulation of a variety of processes [7, 21, 37]. The complex features and functions of this family have been studied extensively in the model herbaceous plants* Arabidopsis* and rice and in the woody plant poplar. Characterization of the* WRKY* genes in grape (*Vitis *spp.) would facilitate a broader understanding of this gene superfamily. In this study, 80* WRKY* genes were characterized. The lengths of these sequences were highly varied implying a high degree of complexity among the* VvWRKY* genes.

The* WRKY* genes can be divided into three main groups based on their structural features. The Groups Ib and II* WRKY* genes are likely to have evolved from Group Ia* WRKY* genes through loss of the N-terminal WRKY domain. Replacement of the conserved His residue in the metal-chelating zinc finger motif with a Cys residue may have led to the evolution of Group III* WRKY* genes [33]. The similar numbers of* Arabidopsis*, rice, poplar, and grape* WRKY* genes in Groups IIa and IIb suggest that all* VvWRKY* genes belonging to these subgroups have been identified, but there were only Group Ia in grape (Table 3). Fewer Group III* WRKY *genes have been identified in grape compared to in* Arabidopsis* and rice, close to the poplar quantitatively, implying that the number of* VvWRKY* genes in this group has either declined over evolutionary time or was underestimated in our analysis. Of the* VvWRKY* genes, 67.9% belong to Group II and 9.7% to Group III. This distribution is more similar to that of the PtWRKY family than the AtWRKY or OsWRKY family, indicating a similar evolutionary history between grape and poplar (Table 3). A phylogenetic tree was constructed based on 58 Group III genes from* Arabidopsis*, rice, poplar, and grape.

The* WRKY* genes may act as a regulatory node that plays a crucial role in responses to abiotic stresses or in stress-induced defense signaling pathways [17]. Plant response to pathogens is regulated by multiple signal transduction pathways, in which SA functions as key signaling molecules [15, 38]. Considerable effort has been directed toward elucidating the regulatory network controlling expression of SA-inducible genes. AtWRKY70 of* Arabidopsis thaliana* was in the SA-signal transduction pathway leading to PR gene expression [15]. Many of the* WRKY* genes were responsive to fungal infection and SA treatment, leading us to suspect that they may also play a regulatory role in the establishment of disease tolerance and in the SA signal transduction pathway in grape [34, 39, 40]. Expression analysis of* AtWRKY *genes in* Arabidopsis *showed that almost 70% are differentially regulated in response to pathogen infection and SA treatment, suggesting that the major role of* WRKY *genes in flowering plants is to mediate defense responses [41]. Details of the roles of the* WRKY *genes in defending dicotyledonous plants against pathogens can be found in three excellent reviews [4, 7, 42]. In our results,* WRKY *genes in grape showed that almost 57% (16 genes) were differentially regulated in response to both pathogen infection and SA treatment. It was lower than* Arabidopsis.* We speculated that the reason was that* WRKY* genes belong to* V. vinifera* PN40024 genomic sequence, and the test material was* V. vinifera *“Pinor,” which was susceptible cultivars Europe grape.

## Figures and Tables

**Table 1:** Members of the grape WRKY superfamily of transcription factors.

Name	Protein^a^	Chr	Deduced polypeptide			Organism	Group	Domain	
			Length (aa)	PI	MW (kDa)			Family name	Pattern
VvWRKY 1-1	GI:50953501	∗	151	9.61	17.7	*Vitis vinifera *	IIc	1-1	C-X_4_-C-X_23_-HXH
VvWRKY 1-2	GI:50953502	∗	297	9.67	17.7	*Vitis vinifera *	IIc	1-2	C-X_4_-C-X_23_-HXH
VvWRKY 2-1	GI:359476618	4	700	6.52	75.9	*Vitis vinifera *	I-NTWD	2-1N	C-X_4_-C-X_22_-HXH
							I-CTWD	2-1C	C-X_4_-C-X_23_-HXH
VvWRKY 2-2	GI:225463536	19	734	5.7	80.3	*Vitis vinifera *	I-NTWD	2-1N	C-X_4_-C-X_22_-HXH
							I-CTWD	2-2C	C-X_4_-C-X_23_-HXH
VvWRKY 2-3	GI:225469228	∗	536	7.21	58.44	*Vitis vinifera *	I-NTWD	2-3N	C-X_4_-C-X_22_-HXH
							I-CTWD	2-3C	C-X_4_-C-X_23_-HXH
VvWRKY 2-4	GI:359478811	6	336	6.31	21.7	*Vitis vinifera *	IIc	2-4	C-X_4_-C-X_23_-HXH
VvWRKY 3-1	GI:48686707	1	317	7.64	58.3	*Vitis vinifera *	I-NTWD	3-1N	C-X_4_-C-X_22_-HXH
							I-CTWD	3-1C	C-X_4_-C-X_23_-HXH
VvWRKY 3-2	GI:315272006	∗	534	8.22	35.3	*Vitis vinifera *	IIa	3-2	C-X_5_-C-X_23_-HXH
VvWRKY 4	GI:315272008	∗	625	8.71	34.4	*Vitis vinifera *	IIa	4	C-X_5_-C-X_25_-HXH
VvWRKY 6-1	GI:359485613	12	753	6.48	64.1	*Vitis vinifera *	IIb	6-1	C-X_5_-C-X_23_-HXH
VvWRKY 6-2	GI:225444291	10	535	6.04	57.5	*Vitis vinifera *	IIb	6-2	C-X_5_-C-X_23_-HXH
VvWRKY 7-1	GI:225458699	18	347	9.36	38.1	*Vitis vinifera *	IId	7-1	C-X_5_-C-X_23_-HXH
VvWRKY 7-2	GI:225438803	7	535	9.57	36.6	*Vitis vinifera *	IId	7-2	C-X_5_-C-X_23_-HXH
VvWRKY 9	GI:225447777	12	798	5.16	52.4	*Vitis vinifera *	IIb	9	C-X_5_-C-X_23_-HXH
VvWRKY 11-1	GI:225466161	4	338	9.49	36.6	*Vitis vinifera *	IId	11-1	C-X_5_-C-X_23_-HXH
VvWRKY 11-2	GI:225445976	11	297	9.73	32.4	*Vitis vinifera *	IId	11-2	C-X_5_-C-X_23_-HXH
VvWRKY 11-3	GI:262091438	∗	297	9.73	32.4	*Vitis vinifera *	IId	1-3	C-X_5_-C-X_23_-HXH
VvWRKY 12-1	GI:225453346	15	228	7.68	26.1	*Vitis vinifera *	IIc	12-1	C-X_4_-C-X_23_-HXH
VvWRKY 12-2	GI:225453345	15	228	7.8	26.1	*Vitis vinifera *	IIc	12-2	C-X_4_-C-X_23_-HXH
VvWRKY 13-1	GI:359472522	1	305	5.8	33.4	*Vitis vinifera *	IIc	13-1	C-X_4_-C-X_23_-HXH
VvWRKY 13-1	GI:359472523	1	305	5.81	33.4	*Vitis vinifera *	IIc	13-1	C-X_4_-C-X_23_-HXH
VvWRKY 14	GI:225444177	10	438	5.16	47.5	*Vitis vinifera *	IIe	14	C-X_5_-C-X_23_-HXH
VvWRKY 18	GI:359476150	4	261	9.1	28.9	*Vitis vinifera *	IIa	18	C-X_5_-C-X_23_-HXH
VvWRKY 20-1	GI:359496861	∗	604	6.01	65.7	*Vitis vinifera *	I-NTWD	20-1N	C-X_4_-C-X_22_-HXH
							I-CTWD	20-1C	C-X_4_-C-X_23_-HXH
VvWRKY 20-2	GI:359494165	19	580	6.27	63.4	*Vitis vinifera *	I-NTWD	20-2N	C-X_4_-C-X_23_-HXH
							I-CTWD	20-2C	C-X_4_-C-X_23_-HXH
VvWRKY 20-3	GI:225447598	12	407	5.02	44.8	*Vitis vinifera *	I-NTWD	20-3N	C-X_4_-C-X_22_-HXH
							I-CTWD	20-3C	C-X_4_-C-X_23_-HXH
VvWRKY 20-4	GI:359496860	12	514	6.6	56.2	*Vitis vinifera *	I-NTWD	20-4N	C-X_4_-C-X_23_-HXH
							I-CTWD	20-4C	C-X_4_-C-X_23_-HXH
VvWRKY 20-5	GI:359494164	19	595	6.8	65	*Vitis vinifera *	I-NTWD	20-5N	C-X_4_-C-X_23_-HXH
							I-CTWD	20-5C	C-X_4_-C-X_22_-HXH
VvWRKY 20-6	GI:359485885	12	407	4.8	44.8	*Vitis vinifera *	I-NTWD	20-6N	C-X_4_-C-X_23_-HXH
							I-CTWD	20-6C	C-X_4_-C-X_23_-HXH
VvWRKY 21	GI:225437249	7	340	9.84	38	*Vitis vinifera *	IId	21	C-X_5_-C-X_23_-HXH
VvWRKY 22-1	GI:359480165	7	233	6.06	26.8	*Vitis vinifera *	IIe	22-1	C-X_5_-C-X_23_-HXH
VvWRKY 22-2	GI:225454298	15	348	5.73	38	*Vitis vinifera *	IIe	22-2	C-X_5_-C-X_23_-HXH
VvWRKY 22-3	GI:225426142	2	331	5.71	36.6	*Vitis vinifera *	IIe	22-3	C-X_5_-C-X_23_-HXH
VvWRKY 22-4	GI:225464629	∗	166	4.7	18.4	*Vitis vinifera *	IIe	22-4	C-X_5_-C-X_23_-HXH
VvWRKY 23	GI:225437606	7	302	6.74	33.8	*Vitis vinifera *	IIc	23	C-X_4_-C-X_23_-HXH
VvWRKY 24	GI:359489647	15	165	9.61	19	*Vitis vinifera *	IIc	24	C-X_4_-C-X_23_-HXH
VvWRKY 28-1	GI:225463412	10	319	6.76	35	*Vitis vinifera *	IIc	28-1	C-X_4_-C-X_23_-HXH
VvWRKY 28-2	GI:225446835	12	311	6.92	34.8	*Vitis vinifera *	IIc	28-2	C-X_4_-C-X_23_-HXH
VvWRKY 30	GI:40846374	∗	349	5.02	39.3	*Vitis aestivalis *	III	30	C-X_7_-C-X_23_-HTC
VvWRKY 32-1	GI:225445873	11	499	6.12	53.8	*Vitis vinifera *	I-NTWD	32-1N	C-X_4_-C-X_22_-HXH
							I-CTWD	32-1C	C-X_4_-C-X_23_-HXH
VvWRKY 32-2	GI:225430477	4	475	8.47	52.6	*Vitis vinifera *	I-NTWD	32-2N	C-X_4_-C-X_22_-HXH
							I-CTWD	32-2C	C-X_4_-C-X_23_-HXH
VvWRKY 33-1	GI:225439574	8	552	7.29	61	*Vitis vinifera *	I-NTWD	33-1N	C-X_4_-C-X_22_-HXH
							I-CTWD	33-1C	C-X_4_-C-X_23_-HXH
VvWRKY 33-2	GI:225434421	6	603	6.42	66.3	*Vitis vinifera *	I-NTWD	33-2N	C-X_4_-C-X_22_-HXH
							I-CTWD	33-2C	C-X_4_-C-X_23_-HXH
VvWRKY 40-1	GI:225443178	9	311	5.16	52.4	*Vitis vinifera *	IIa	40-1	C-X_5_-C-X_25_-HXH
VvWRKY 40-2	GI:225430340	4	317	8.22	35.3	*Vitis vinifera *	IIa	40-2	C-X_5_-C-X_23_-HXH
VvWRKY 41	GI:225426000	2	342	6.05	38.6	*Vitis vinifera *	III	41	C-X_7_-C-X_23_-HTC
VvWRKY 42	GI:359494147	19	511	9.2	55.1	*Vitis vinifera *	IIb	42	C-X_5_-C-X_23_-HXH
VvWRKY 44	GI:225439779	8	477	8.84	52.3	*Vitis vinifera *	I-NTWD	44C	C-X_4_-C-X_22_-HXH
							I-CTWD	44N	C-X_4_-C-X_23_-HXH
VvWRKY 45	GI:225451489	14	182	9.41	20.8	*Vitis vinifera *	IIc	45	C-X_4_-C-X_23_-HXH
VvWRKY 46	GI:225454483	15	349	5.01	39.2	*Vitis vinifera *	III	46	C-X_7_-C-X_23_-HTC
VvWRKY 47	GI:225437767	7	505	8.03	54.7	*Vitis vinifera *	IIb	47	C-X_5_-C-X_23_-HXH
VvWRKY 48	GI:225432004	5	309	5.72	34.4	*Vitis vinifera *	IIc	48	C-X_4_-C-X_23_-HXH
VvWRKY 49	GI:225440394	8	299	5.16	52.4	*Vitis vinifera *	IIc	49	C-X_4_-C-X_23_-HXH
VvWRKY 50	GI:225429590	4	166	5.2	18.9	*Vitis vinifera *	IIc	50	C-X_4_-C-X_23_-HXH
VvWRKY 51-1	GI:359476460	4	136	9.39	15.8	*Vitis vinifera *	IIc	51-1	C-X_4_-C-X_23_-HXH
VvWRKY 51-2	GI:359480857	7	149	9.07	17.2	*Vitis vinifera *	IIc	51-2	C-X_4_-C-X_23_-HXH
VvWRKY 51-3	GI:225466167	4	191	5.58	21.5	*Vitis vinifera *	IIc	51-3	C-X_4_-C-X_23_-HXH
VvWRKY 51-4	GI:359480856	7	193	7.1	21.5	*Vitis vinifera *	IIc	51-4	C-X_4_-C-X_23_-HXH
VvWRKY 53	GI:359490533	16	364	5.45	40	*Vitis vinifera *	III	53	C-X_7_-C-X_23_-HTC
VvWRKY 57-1	GI:225423515	1	305	5.62	33.4	*Vitis vinifera *	IIc	57-1	C-X_4_-C-X_23_-HXH
VvWRKY 57-2	GI:225425363	1	189	9.4	21.3	*Vitis vinifera *	IIc	57-2	C-X_4_-C-X_23_-HXH
VvWRKY 65-1	GI:225443744	10	278	5.14	3.9	*Vitis vinifera *	IIe	65-1	C-X_5_-C-X_23_-HXH
VvWRKY 65-2	GI:225446682	12	244	5.41	26.4	*Vitis vinifera *	IIe	65-2	C-X_5_-C-X_23_-HXH
VvWRKY 65-3	GI:359485307	12	244	5.4	26.4	*Vitis vinifera *	IIe	65-3	C-X_5_-C-X_23_-HXH
VvWRKY 55	GI:225448719	13	364	5.97	40.3	*Vitis vinifera *	III	55	C-X_7_-C-X_23_-HTC
VvWRKY 70-1	GI:225448721	13	313	5.45	35.3	*Vitis vinifera *	III	70-1	C-X_7_-C-X_23_-HTC
VvWRKY 70-2	GI:225439707	8	322	5.49	36.6	*Vitis vinifera *	III	70-2	C-X_7_-C-X_23_-HTC
VvWRKY 72-1	GI:359491334	17	611	7.9	65.7	*Vitis vinifera *	IIb	72-2	C-X_5_-C-X_23_-HXH
VvWRKY 72-2	GI:359488978	14	755	5.85	59.7	*Vitis vinifera *	IIb	72-2	C-X_5_-C-X_23_-HXH
VvWRKY 72-3	GI:359473376	1	547	5.92	59.7	*Vitis vinifera *	IIb	72-3	C-X_5_-C-X_23_-HXH
VvWRKY 74	GI:225463956	14	362	9.68	41.3	*Vitis vinifera *	IId	74	C-X_5_-C-X_23_-HXH
VvWRKY 75	GI:225456341	17	151	9.67	17.7	*Vitis vinifera *	IIc	75	C-X_4_-C-X_23_-HXH
VtWRKY5	GI:183979104	∗	529	7.72	57.7	*Vitis thunbergii *	I-NTWD	Vt5N	C-X_4_-C-X_22_-HXH
							I-CTWD	Vt5C	C-X_4_-C-X_23_-HXH
VtWRKY7	GI:183979108	∗	603	6.42	66.3	*Vitis thunbergii *	I-NTWD	Vt7N	C-X_4_-C-X_22_-HXH
							I-CTWD	Vt7C	C-X_4_-C-X_23_-HXH
VtWRKY11	GI:183979106	∗	338	9.49	36.6	*Vitis thunbergii *	IId	Vt11	C-X_5_-C-X_23_-HXH
VaWRKY4	GI:40060529	∗	311	8.71	34.4	*Vitis aestivalis *	IIa	Va4	C-X_5_-C-X_23_-HXH
VpWRKY1	GI:263199372	∗	322	5.58	36.5	*Vitis pseudoreticulata *	III	Vp1	C-X_7_-C-X_23_-HTC
VpWRKY2	GI:290894627	∗	499	6.23	53.9	*Vitis pseudoreticulata *	I-NTWD	Vp2N	C-X_4_-C-X_22_-HXH
							I-CTWD	Vp2C	C-X_4_-C-X_23_-HXH
VpWRKY3	GI:345104746	∗	319	7.67	35.5	*Vitis pseudoreticulata *	IIa	Vp3	C-X_5_-C-X_23_-HXH
VbcWRKY50	GI:163914201	∗	127	9.4	14.3	*Vitis* hybrid cultivar	IId	Vbc50	C-X_5_-C-X_23_-HXH

^a^GenBank protein number

Chr.: chromosome; ORF: open reading frame; *chromosome unknown.

**Table 2:** Motif sequences.

Motif	Width	Best possible match
1	31	GCPVRKHVERCSEDPSMVITTYEGEHNHPVP
2	21	ILDDGYRWRKYGQKVIKGNPY
3	41	EKPSEDGYNWRKYGQKQVKGSEYPRSYYKCTHPNCPVKKKV
4	8	PRSYYRCT
5	29	EEINKKDKKKGHKKIREPRFCFQTRSEVD
6	31	ERSHDGQITEIIYKGTHNHPKPQPNRRYAVG
7	50	YRQMRPAKLPIARSPCFTIPPGLSPTCLLDSPVFLSNMKVEPSPTTGTFP
8	41	ETGVLVEELNRMNEENKKLREMLEIMCENYNALQMHLMELM
9	29	FLVEQMTAAITKDPNFTAALAAAISGIIL
10	29	SGRCHCSKRRKMRVKRTIRVPAISSKIAD
11	29	QMASMMCPISMSTPFPTITLDLTKPTSFS
12	29	INHFDCREITDYTVSKFKRVISILNRTGH
13	41	NRNNIHGSVGNNTYSTSMDDIFRKKREETDKIKFRRVYYIT
14	29	PAATAMASTTSAAASMLLSGSMTSQDGLM
15	15	GDEDDEDEPDSKRWK
16	29	GFSKMDEQIAIQEAASAGLKSMEHLIRLL
17	50	DPNGHANFQENPELGSQGQMGNLNKPNEGLPAYSLPGMDQETTQAMPLHL
18	24	WEHKTLINELTQGREMAKQLKIHL
19	41	TSMESVPIEVDYDKLQQRQHFNIGVQASQSEQKETNPIIVV
20	41	NWMAASLDLNANPLRLFDDTPKKEVQDDFTGLGLKVVSLKE

**Table 3:** Numbers of the various types of *WRKY* genes in *Arabidopsis*, rice, poplar, and grape.

Group	Subgroup	Gene number				
		AtWRKY^a^	OsWRKY^a^	PtWRKY^a^	VvWRKY^a^	VxWRKY^b^
I		32	34	50	16	3
	Ia	14	14	23	16	3
	Ib	18	20	27		

II		26	30	44		
	IIa	3	4	5	5	2
	IIb	8	8	9	7	
	IIc	7	7	13	22	
	IId	8	11	13	7	2
	IIe			4	8	

III		14	36	10	7	1

Total		72	100	104	72	8

^a^According to Wu et al. [20] and He et al. [21].

^b^ *Vitis thunbergii*, *Vitis thunbergii*, *Vitis pseudoreticulata*, and *Vitis* hybrid cultivar.
